# Predicting Resistance by Mutagenesis: Lessons from 45 Years of MBC Resistance

**DOI:** 10.3389/fmicb.2016.01814

**Published:** 2016-11-15

**Authors:** Nichola J. Hawkins, Bart A. Fraaije

**Affiliations:** Biological Chemistry and Crop Protection, Rothamsted ResearchHarpenden, UK

**Keywords:** fungicide resistance, plant pathogens, benzimidazoles, mutagenesis, *in vitro* selection, predictability, fitness penalties, functional constraints

## Abstract

When a new fungicide class is introduced, it is useful to anticipate the resistance risk in advance, attempting to predict both risk level and potential mechanisms. One tool for the prediction of resistance risk is laboratory selection for resistance, with the mutational supply increased through UV or chemical mutagenesis. This enables resistance to emerge more rapidly than in the field, but may produce mutations that would not emerge under field conditions. The methyl benzimidazole carbamates (MBCs) were the first systemic single-site agricultural fungicides, and the first fungicides affected by rapid evolution of target-site resistance. MBC resistance has now been reported in over 90 plant pathogens in the field, and laboratory mutants have been studied in nearly 30 species. The most common field mutations, including β-tubulin E198A/K/G, F200Y and L240F, have all been identified in laboratory mutants. However, of 28 mutations identified in laboratory mutants, only nine have been reported in the field. Therefore, the predictive value of mutagenesis studies would be increased by understanding which mutations are likely to emerge in the field. Our review of the literature indicates that mutations with high resistance factors, and those found in multiple species, are more likely to be reported in the field. However, there are many exceptions, possibly due to fitness penalties. Whether a mutation occurred in the same species appears less relevant, perhaps because β-tubulin is highly conserved so functional constraints are similar across all species. Predictability of mutations in other target sites will depend on the level and conservation of constraints.

## Introduction

The loss of effective fungicide classes due to the evolution of resistance in key target pathogens is a major threat to crop protection. The methyl benzimidazole carbamates (MBCs), or benzimidazoles, were the first single-site fungicides, and the first cases of MBC resistance were reported soon after their introduction. This was followed by the introduction of, and subsequent emergence of resistance to, the 2-aminopyrimidine mildewicides; the phenylamide oomyceticides; the demethylation inhibitor (DMI) fungicides, including azoles; and the Quinone outside Inhibitor (QoI) fungicides, or strobilurins (Lucas et al., 2015). In contrast, cases of resistance against multi-site inhibitors remain rare (Grimmer et al., 2014).

With the recent introduction of new succinate dehydrogenase inhibitors (SDHIs), it was realized that resistance would be a risk. Consequently, mutagenesis and laboratory selection experiments were carried out to assess the resistance risk and possible mechanisms in advance of resistance emerging in the field (Fraaije et al., 2012; Scalliet et al., 2012). These experiments use UV irradiation as a mutagen, increasing the mutational supply, coupled with strong selection from a discriminatory dose of fungicide within the growth medium.

These laboratory selection experiments rapidly produced resistant mutants carrying a range of target-site mutations, correlated with a range of resistance factors. However, questions remained as to which of these mutations would actually emerge in the field: whether a single highly resistant genotype would dominate as seen with the QoIs; or whether the range of mutations and resistance factors gave cause for optimism that resistance may emerge in the slower, step-wise fashion seen with the azoles.

We consider mutagenesis studies carried out with MBC selection in the light of over 45 years of field resistance reports, comparing the mutations produced in the laboratory with those that have actually been reported in the field.

## MBC Resistance

The first published case of MBC resistance was in cucurbit powdery mildew in 1969 (Schroeder and Provvidenti, 1969), followed by Botrytis in grapevine in 1971 (Ehrenhardt et al., 1973), and cereal powdery mildew in 1973 (Vargas, 1973). Resistance has now been reported in over 90 different plant pathogens in the field (Fungicide Resistance Action Committee, 2013).

Since the introduction of MBCs and the first reports of field resistance, mutagenesis studies have also been carried out. Initially these studies were carried out in the model fungi *Saccharomyces cerevisiae* (Thomas et al., 1985), *Neurospora crassa* (Borck and Braymer, 1974; Orbach et al., 1986; Fujimura et al., 1992), and *Aspergillus nidulans* (Jung and Oakley, 1990; Jung et al., 1992), in order to confirm the mode of action and resistance mechanism. Subsequent studies have sought to determine the potential for MBC resistance in other plant pathogen species (Wheeler et al., 1995; Albertini et al., 1999; Ziogas et al., 2009), clinical pathogens (Cruz and Edlind, 1997), and phytopathogen biocontrol agents (Olejnikova et al., 2010).

When field resistance was first reported (Schroeder and Provvidenti, 1969), the resistance mechanism was unknown. Laboratory mutants in model species were then used in protein binding studies (Davidse and Flach, 1977) and protein electrophoresis (Sheir-Neiss et al., 1978), demonstrating reduced fungicide binding and altered electrophoretic properties of the target protein from resistant mutants, identified as tubulin and specifically β-tubulin. This was followed by gene cloning (Orbach et al., 1986) and sequencing (Thomas et al., 1985; Fujimura et al., 1990) of *β-tubulin* from resistant mutants, identifying the individual mutations responsible. Some two decades after the first reports of field resistance, Koenraadt et al. (1992) reported target-site mutations in MBC-resistant field isolates of plant pathogens. The *β-tubulin* mutations responsible for MBC resistance in field isolates have now been published for 29 fungal species.

## Lab and Field

Mutagenesis studies have undoubtedly proven useful in mode of action studies, identifying the target sites of fungicides and the gene that would be involved in target-site resistance. However, mutagenesis studies are sometimes also used to predict which mutations within that gene may confer resistance in the field. We analyse the accuracy of such predictions in the case of MBC resistance.

**FIGURE 1:**
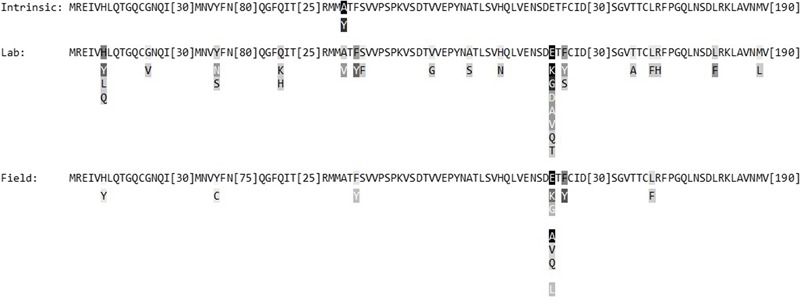
**Summary of key β-tubulin amino acid substitutions in intrinsically resistant fungal species, and resistant laboratory mutants and field isolates of other fungal species.** Top lines: wild-type consensus sequence; darker shading indicates residues at which substitutions occur in more species. Square brackets: omitted residues (no substitutions). Letters under sequences: substituting amino acids in resistant mutants/isolates; darker shading indicates substitutions occurring in more species.

The first *β-tubulin* mutations reported in field isolates resulted in E198A, E198K, E198V, or F200Y amino acid substitutions (Koenraadt et al., 1992). These mutations have since been reported in a range of other pathogens, along with a further eight non-synonymous mutations. The most-reported substitutions across species are E198A in 20 species, and F200Y in 14 species. E198K has been reported in 12 species; E198G in six; L240F in four; F167Y and E198L in three species each; E198Q and E198V in two species; and H6Y, Y50C and Q73R, each in a single species (**Figure 1**; Supplementary Table 1). Y167 has also been reported in the intrinsically resistant *Cochliobolus heterostrophus* and *Stemphylium* species (Gafur et al., 1998; Huang et al., 2013).

Methyl benzimidazole carbamate-resistant laboratory mutants have been reported in nearly 30 fungal species (Fungicide Resistance Action Committee, 2013). The *β-tubulin* mutations in those mutants have been published for 15 of those species (Supplementary Table 1). The most common mutations in laboratory mutants are at codon 198, as they are for field isolates. However, in laboratory mutants, the substitutions E198K and E198G are found in more species than E198A. F167Y and H6Y are also reported in laboratory mutants in multiple species, and have been found in the field as well. However, many other laboratory mutations have not yet been reported in the field: of 28 reported amino acid changes in laboratory mutants, only nine have been reported in the field, leaving 19 substitutions predicted by mutagenesis studies that have yet to be found in field isolates. In contrast, only a further three non-synonymous changes have been reported in field isolates without having been identified in laboratory mutants.

Therefore, laboratory mutagenesis studies can identify a range of potential mutations, of which a subset may occur in the field. This raises the question of why some mutations occur in the field and others do not, and whether the predictive power of mutagenesis studies can be improved by assessing which mutations are most likely to be reported in field isolates.

## Resistance Factors

One possibility is that the most resistant mutants will be reported in the field. This may be due to a higher emergence rate of such mutations due to stronger positive selection by fungicides, or due to reporting bias since highly resistant isolates are more likely to result in control failures prompting further investigation.

**FIGURE 2:**
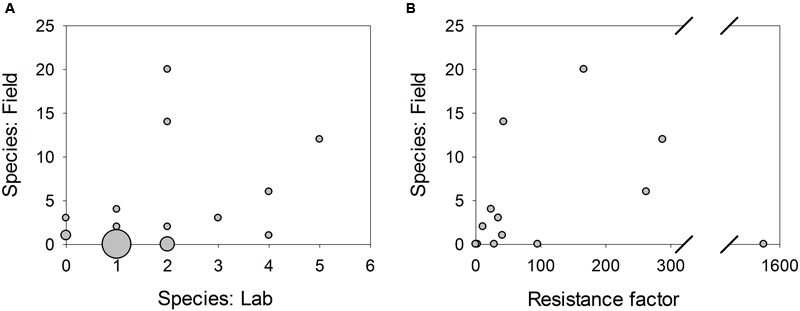
**Scatter plots showing the number of species in which a β-tubulin substitution has been reported in MBC-resistant field isolates, against **(A)** the number of species in which that mutation has been reported in laboratory isolates; **(B)** the mean resistance factor of mutants with that substitution based on published sensitivity data.** Symbol area is proportional to number of substitutions.

Sensitivity data were included in 12 published mutagenesis studies. Sensitivity data reported as MIC or EC_50_ values were converted to resistance factors. Geometric means of resistance factors were calculated where multiple data were available for a single mutation. We assumed general positive cross-resistance between MBC fungicides where different fungicides were tested in different species but excluded data for N-phenyl-carbamates such as diethofencarb. The number of species in which each mutation has been reported in the field was plotted against the average resistance factor for that mutation (**Figure 2**).

Out of those mutations for which laboratory mutant sensitivity data were available, four of the top six mutations by resistance factors were the top four mutations reported in the most species in the field (E198E, E198G, E198K, and F200Y). Mutations with lower resistance factors have been reported in fewer or no species in the field, which may be due to insufficient selective pressure in the field, or insufficient resistance to prompt investigation. However, there is scatter within the higher and lower groups and there is no significant correlation overall.

Two substitutions with high resistance factors but not reported in the field are G13V and S168F, both found in *Trichoderma harzianum* (Li et al., 2013). The S168F mutant had the highest resistance factor with a 1572-fold increase in EC_50_ compared to the wild-type, and the G13V mutant had a 96-fold increase. When mutants are found in the laboratory and not in the field, fitness penalties are often invoked as a possible explanation. Both of those mutants appeared to exhibit normal growth in culture, although fitness was not tested under field conditions. However, since both mutations were reported in the same species and within the same study, it is possible that these data points reflect problems with comparing data from different experimental systems and assay methods.

In other studies, mutants have been found to carry fitness penalties, often in the form of increased temperature sensitivity. Microtubule dynamics within a cell require both the polymerisation and depolymerisation of tubulin. Therefore, there will be fitness penalties both for β-tubulin alterations causing reduced binding affinity, especially at higher temperatures; and for alterations causing stronger binding, especially in lower temperatures. In *S. cerevisiae*, Thomas et al. (1985) reported six cold-sensitive mutants including R241H, and three cold-sensitive mutants; Li et al. (1996) report that F167Y also confers cold-sensitivity. In *Fusarium moniliforme*, Y50N mutants were fungicide-sensitive at low temperatures but could grow in the absence of fungicides (Yan and Dickman, 1996). In *B. bassiana*, Zou et al. (2006) reported mutant haplotypes with up to six *β-tubulin* mutations with a range of sensitivity levels, with reduced thermal tolerance in the more resistant mutants. In *A. nidulans*, benA33 mutants were cold-sensitive due to microtubule hyperstability (Oakley and Morris, 1981; Davidse, 1986), whereas mutations Y50N, Y50S, Q134K, and M257L were associated with heat-sensitivity (Koenraadt et al., 1992). However, Weatherbee and Morris (1984) describe “revertants” growing at high temperatures, some of which retained MBC-resistance, indicating that compensatory mutations are possible. Furthermore, Ma et al. (2003) report temperature sensitivity in field isolates of *Monilinia fructicola* with H6Y isolates cold-sensitive and E198A isolates heat-sensitive, and Trkulja et al. (2013) report that MBC-resistance is cold-sensitive in F167Y isolates of *Cercospora beticola*, demonstrating that fitness costs involving temperature-sensitivity may not prevent the emergence of a mutation in the field.

## Occurrence in Different Species

Another hypothesis was that a mutation having been selected repeatedly in mutagenesis studies across different species would indicate that it is more likely to be found in the field. The number of species in which each mutation has been reported in the field was plotted against the number of species in which that mutation had been generated in laboratory mutants.

There was no overall correlation between the number of species in which a mutation was reported in the field and in laboratory mutants. However, mutations found in multiple species in the laboratory were more likely to be found in the field at all. All four mutations reported in laboratory mutants of three or more species (E198K, E198G, F167Y, and H6Y) have been reported in at least one species in the field, compared to three out of seven mutations each reported in laboratory mutants of two species; just two out of 17 mutations reported in laboratory mutants of a single species; and three out of the 8503 potential amino acid substitutions not reported in laboratory mutants.

A further question is whether mutations are more likely to emerge in the field in a given species if they have occurred in laboratory mutants of that species, compared to having occurred in mutants of any species. There are only six species for which *β-tubulin* mutations have been published both from laboratory mutagenesis studies and from MBC-resistant field isolates. Within those six species, an average of 2.67 mutations per species have been reported in laboratory mutants and 2.67 mutations per species in field isolates, of which 0.67 mutations are found in both laboratory and field strains of that same species. Over the six species as a whole, a total of 14 mutations were reported in laboratory mutants; of the 2.67 mutations per species and seven mutations in total in field isolates, all had been found in laboratory mutants.

Therefore, if data are limited to the same species, 25% of laboratory mutations were found in the field, 25% of field mutations were found in laboratory and 14% of the total mutations were found both in laboratory mutants and in field isolates. If field mutations within each species are compared to laboratory mutations across the six species, only 19% of laboratory mutations are found in field but 100% of field mutations were found in the laboratory, with 19% of total mutations found in both laboratory and field strains. If laboratory and field mutations are considered across the six species, 50% of laboratory mutations are found in field isolates, 100% of field mutations had been reported in laboratory mutants, with 50% total overlap.

These figures appear mainly to reflect sampling effects. If more species are considered, there is more coverage of all potential mutation space, leading to more overlap between laboratory and field mutations. The species in which the mutations occurred appears to be less relevant: this is likely to be due to the conserved nature of the target site. Fungal β-tubulin is a highly conserved protein, and therefore its evolution in response to fungicide selection is likely to be determined by similar functional constraints across species. It remains to be seen whether this apparent species-independence is generally true for mutagenesis studies, or whether other molecular targets have more lineage-specific constraints.

This has implications as to whether it is useful to carry out mutagenesis in a new target species if similar studies have already been carried out in other fungi. These results suggest that mutagenesis in additional species would be useful since it increases the total sampling of mutations, but for conserved targets like β-tubulin it could be more useful to generate plenty of mutants in an easy-to-grow, readily mutated laboratory model than to struggle with a less tractable target pathogen.

## Mutated Codon Positions

Finally, we consider whether the predictive power of mutagenesis studies is higher in terms of the codons at which substitutions will occur than for the specific substitutions. For specific substitutions, 24% of laboratory mutations have been reported in the field and 42% of field mutations had been reported in the laboratory, with 29% total in overlap. For codon position only, 26% of residues altered in laboratory mutants were also altered in field isolates and 46% of residues altered in field isolates had also been altered in laboratory mutants, with 33% total overlap. Therefore, by considering only the codon and not the specific substitution, the overlap rate is marginally higher. However, substantially less of the total possible mutational space is excluded. Of 449 codons in the fungal *β-tubulin* consensus sequence, 18 have reported mutations, which is approximately 1 in 40. Out of 8531 possible substitutions, 31 have been reported, which is approximately 1 in 500. Therefore, considering codons only leads to a marginally lower error rate but a substantially lower information content.

The main exception to this is where mutagenesis was carried out using chemical mutagens that have a substitution bias and so are more likely to introduce a different substitution at the same codon. In *Colletotrichum gloeosporioides*, E198K was reported in laboratory mutants whereas E198A is more common in the field, since the mutagen used, EMS, introduces A:T nucleotide transitions whereas E198A requires an A:C transversion (Buhr and Dickman, 1994; Lucas et al., 2015). In contrast, UV mutagenesis produces a range of mutations, with all 12 possible nucleotide substitutions found within MBC-selected laboratory mutants.

## Future Prospects

Following the MBCs, further single-site fungicide classes have been introduced and resistance has subsequently emerged. In the case of the QoIs, the picture is somewhat simpler. A single mutation, G143A, is responsible for the majority of field resistance; an alternative mutation, F129L, occurs mostly in species where the mutation encoding G143A would prevent the correct splicing of an intron after codon 143; and a third alternative, G137R, at very low frequency in a single species (Sierotzki et al., 2007). In contrast, the picture for azoles is far more complex, with over 30 reported mutations in *Zymoseptoria tritici* alone, combining in multi-mutation haplotypes (Cools and Fraaije, 2013). This is further complicated by the incomplete cross-resistance between different compounds within the DMI group (Cools et al., 2013), so a mutagenesis study selecting with one azole may produce different mutations from selection with another.

Therefore, the use of mutagenesis has been limited for both fungicide classes, perhaps for opposite reasons: in QoIs, because the rapid emergence of a single, overwhelmingly prevalent mutation has made such studies unnecessary; and in azoles, far more extensive studies would be required to produce meaningful results. For the QoIs, Geier et al. (1992) report two cytochrome *b* mutations including G137R in yeast mutants and Malandrakis et al. (2006) report six different mutations including G143A and F129V in laboratory mutants of *C. beticola*. For the azoles, Chen et al. (2012) identified the CYP51 substitution Y136F, which has been reported in several species in the field, in laboratory mutants of *M. fructicola*. The CYP51A substitution G54W has been reported in laboratory mutants of *A. parasiticus* (Doukas et al., 2012) and clinical isolates of *A. fumigatus* (Diaz-Guerra et al., 2003). Krishnan-Natesan et al. (2008) generated *A. flavus* mutants, reporting five mutations in *CYP51A* and four different mutations in *CYP51B*, none known from clinical *Aspergillus* isolates. Doukas et al. (2012) also obtained mutants with an MDR phenotype, as seen in resistant field isolates of plant pathogens with enhanced eﬄux (Hayashi et al., 2001; Leroux and Walker, 2011). Fan et al. (2014) report a proposed loss-of-function mutation in *CYP51B* resulting in over-expression of *CYP51A* in *F. verticillioides*, and Jackson et al. (2003) report sterol desaturase loss-of-function mutants in *S. cerevisiae*.

For the most recently introduced class of ascomycete-active SDHIs, mutagenesis studies were carried out prior to their introduction in the field (Fraaije et al., 2012; Scalliet et al., 2012). Early reports of field resistance (FRAC SDHI Working Group, 2015) are consistent with the pattern noted for MBCs: laboratory mutagenesis generates a wide variety of mutations, of which a subset will be reported in the field. The use of functional genetic approaches such as homologous gene replacement will allow more precise measurements of the impact of each mutation on fungicide sensitivity and of any associated fitness penalties, and it is hoped that a better understanding of the underlying fitness landscape will improve the predictive power of laboratory studies of resistance in future.

## Author Contributions

NH contributed to the conception and design of the work, carried out the literature search and meta-analyses, and drafted the manuscript. BF contributed to the conception and design of the work and critically revised the manuscript.

## Conflict of Interest Statement

The authors declare that the research was conducted in the absence of any commercial or financial relationships that could be construed as a potential conflict of interest.
