# Supplementary material for: Predicting Resistance by Mutagenesis: Lessons from 45 Years of MBC Resistance
Source: Front Microbiol. 2016 Nov 15;7:1814. doi: 10.3389/fmicb.2016.01814 (PMC5108816; doi:10.3389/fmicb.2016.01814)
Supplement: Supplementary file 1 [file Table_1.DOCX]

Supplementary Material

Predicting resistance by mutagenesis: lessons from 45 years of MBC resistance

Nichola J. Hawkins*, Bart A. Fraaije

*** Correspondence:** Corresponding Author: [nichola.hawkins@rothamsted.ac.uk](mailto:nichola.hawkins@rothamsted.ac.uk)

| \| **Codon** \| **Substitution** \| **Species: lab** \| **Species: field** \| **Species: intrinsic** \| \| --- \| --- \| --- \| --- \| --- \| \| 6 \| H-L \| *Aspergillus nidulans* \|  \|  \| \|  \| H-Y \| *Aspergillus nidulans, Parastagonospora nodorum, Trichoderma harzianum, Trichoderma viride* \| *Monilinia fructicola* \|  \| \|  \| H-Q \| *Cryptococcus neoformans* \|  \|  \| \| 13 \| G-V \| *Trichoderma harzianum* \|  \|  \| \| 50 \| Y-N \| *Aspergillus nidulans, Fusarium moniliforme* \|  \|  \| \|  \| Y-S \| *Aspergillus nidulans* \|  \|  \| \|  \| Y-C \|  \| *Cladobotryum dendroides* \|  \| \| 73 \| Q-R \|  \| *Gibberella zeae (with E198L)* \|  \| \| 134 \| Q-K \| *Aspergillus nidulans* \|  \|  \| \|  \| Q-H \| *Beauveria bassiana* \|  \|  \| \| 165 \| A-V \| *Aspergillus nidulans, Neurospora crassa* \|  \|  \| \| 167 \| F-Y \| *Neurspora crassa, Penicillium expansum, Beauvaria bassiana* \| *Gibberella zeae, Cercospora beticola, Penicillium expansum* \| *Cochliobolus heterostrophus*, *Stemphilium spp.* \| \| 168 \| S-F \| *Trichoderma harzianum* \|  \|  \| \| 179 \| V-G \| *Tapesia yallundae* \|  \|  \| \| 185 \| A-S \| *Tapesia yallundae* \|  \|  \| \| 190 \| H-N \| *Tapesia yallundae* \|  \|  \|   **Supplementary Table 1**: Full list of published β-tubulin amino acid substitutions in benzimidazole-resistant fungal laboratory mutants, field isolates and intrinsically-resistant species.   \| 198 \| E-A \| *Botrytis cinerea, Tapesia yallundae* \| *Botrytis cinerea, Penicillium aurantiogriseum, Penicillium expansum, Penicillium puberulum, Venturia inaequalis, Venturia pirina, Tapesia yallundae, Tapesia acuformis, Pyrenopeziza brassicae, Mycosphaerella graminicola, Colletotrichum gloeosporioides, Cercospora beticola, Monilinia laxa, Monilinia fructicola, Colletotrichum cereale, Penicillium expansum, Podosphaera fusca, Sclerotinia sclerotiorum, Cladosporium fulvum, Mycosphaerella fijiensis, Helminthosporium solani* \|  \| \| --- \| --- \| --- \| --- \| --- \| \|  \| E-D \| *Aspergillus nidulans, Tapesia yallundae* \|  \|  \| \|  \| E-Q \| *Aspergillus nidulans* \| *Tapesia acuformis, Gibberella zeae,* \|  \| \|  \| E-G \| *Neurspora crassa, Tapesia yallundae, Botrytis cinerea, Beauvaria bassiana* \| *Rhynchosporium commune, Venturia inaequalis, Tapesia yallundae, Tapesia acuformis, Pyrenopeziza brassicae, Phaeoacremonium aleophilum* \|  \| \|  \| E-K \| *Aspergillus nidulans, Neurspora crassa, Rhynchosporium commune, Colletotrichum gloeosporioides, Beauvaria bassiana* \| *Botrytis cinerea, Monilinia fructicola, Penicillium aurantiogriseum, Penicillium digitatum, Penicillium italicum, Penicillium puberulum, Penicillium viridicatum, Sclerotinia homoeocarpa, Venturia inaequalis, Tapesia yallundae, Colletoctrichum cereale, Penicillium expansum* \|  \| \|  \| E-V \| *Penicillium expansum, Beauvaria bassiana* \| *Botrytis cinerea, Phomopsis obscurans,* \|  \| \|  \| E-L \|  \| *Botrytis cinerea, Gibberella zeae Phaeoacremonium aleophilum* \|  \| \|  \| E-T \| *Tapesia yallundae* \|  \|  \|  \| 200 \| F-Y \| *Aspergillus nidulans, Phomopsis obscurans* \| *Botrytis cinerea, Penicillium aurantiogriseum, Penicillium italicum, Venturia inaequalis, Venturia pirina, R. secalis, T. yallundea, Tapesia acuformis, Gibberella zeae, Pyrenopeziza brassicae, Colletotrichum cereale, Fusarium asiaticum, Sclerotinia sclerotiorum, Cladosporium fulvum* \|  \| \| --- \| --- \| --- \| --- \| --- \| \|  \| F-S \| *Tapesia yallundae* \|  \|  \| \| 237 \| T-A \| *Neurspora crassa* \|  \|  \| \| 240 \| L-F \| *Penicillium expansum* \| *Tapesia yallundae, Pyrenopeziza brassicae, Monilinia laxa, Venturia inaequalis* \|  \| \| 241 \| R-H \| *Saccharomyces cerevisiae* \|  \|  \| \| 250 \| L-F \| *Tapesia yallundae, Neurspora crassa* \|  \|  \| \| 257 \| M-L \| *Aspergillus nidulans* \|  \|  \| |  |  |  |
| --- | --- | --- | --- | --- | --- | --- | --- | --- | --- | --- | --- | --- | --- | --- | --- | --- | --- | --- | --- | --- | --- | --- | --- | --- | --- | --- | --- | --- | --- | --- | --- | --- | --- | --- | --- | --- | --- | --- | --- | --- | --- | --- | --- | --- | --- | --- | --- | --- | --- | --- | --- | --- | --- | --- | --- | --- | --- | --- | --- | --- | --- | --- | --- | --- | --- | --- | --- | --- | --- | --- | --- | --- | --- | --- | --- | --- | --- | --- | --- | --- | --- | --- | --- | --- | --- | --- | --- | --- | --- | --- | --- | --- | --- | --- | --- | --- | --- | --- | --- | --- | --- | --- | --- | --- | --- | --- | --- | --- | --- | --- | --- | --- | --- | --- | --- | --- | --- | --- | --- | --- | --- | --- | --- | --- | --- | --- | --- | --- | --- | --- | --- | --- | --- | --- | --- | --- | --- | --- | --- | --- | --- | --- | --- | --- | --- | --- | --- | --- | --- | --- | --- | --- | --- | --- | --- | --- | --- | --- | --- | --- | --- | --- | --- |

Additional references not cited in text: Burns et al., 1986; Cooley and Caten, 1993; Goldman et al., 1993; Koenraadt and Jones, 1993; Fujimura et al., 1994; Yan and Dickman, 1996; McKay et al., 1998; Baraldi et al., 2003; Cunha and Rizzo, 2003; Ma et al., 2003; Fraaije et al., 2005; Ma et al., 2005; Canas-Gutierrez et al., 2006; Davidson et al., 2006; Maymon et al., 2006; Zou et al., 2006; Wong et al., 2008; Yan et al., 2008; Chen et al., 2009; Liu et al., 2010; Quello et al., 2010; Yin et al., 2010; Young et al., 2010; Zhang et al., 2010; Suga et al., 2011; Malandrakis et al., 2012; Carter et al., 2013; Martin and Martin, 2013; Shi et al., 2013; Trkulja et al., 2013; Vela-Corcia et al., 2014
